# Supplementary material for: Bio-Prospecting of Crude Leaf Extracts from Thirteen Plants of Brazilian Cerrado Biome on Human Glioma Cell Lines
Source: Molecules. 2023 Feb 1;28(3):1394. doi: 10.3390/molecules28031394 (PMC9921846; doi:10.3390/molecules28031394)
Supplement: Supplementary file 1 [file molecules-28-01394-s001.zip › molecules-2073455-supplementary.pdf]

A)

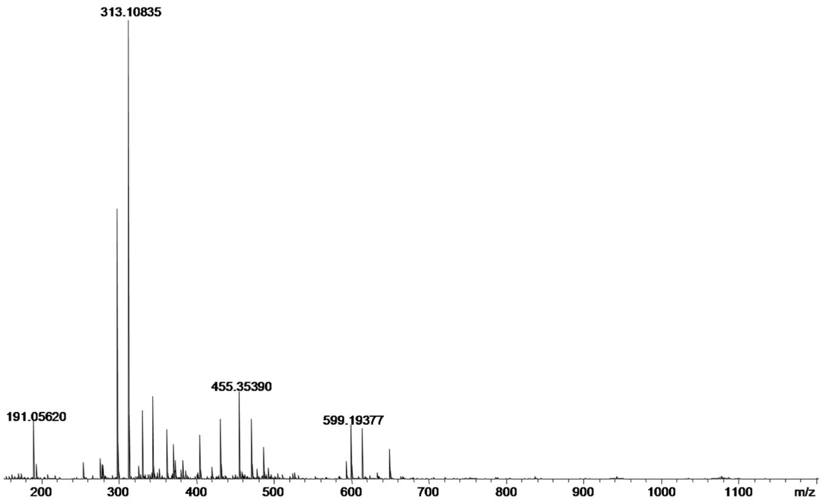
313.10835
455.35390
191.05620
599.19377
200
300
400
500
600
700
800
900
1000
1100
m/z

B)

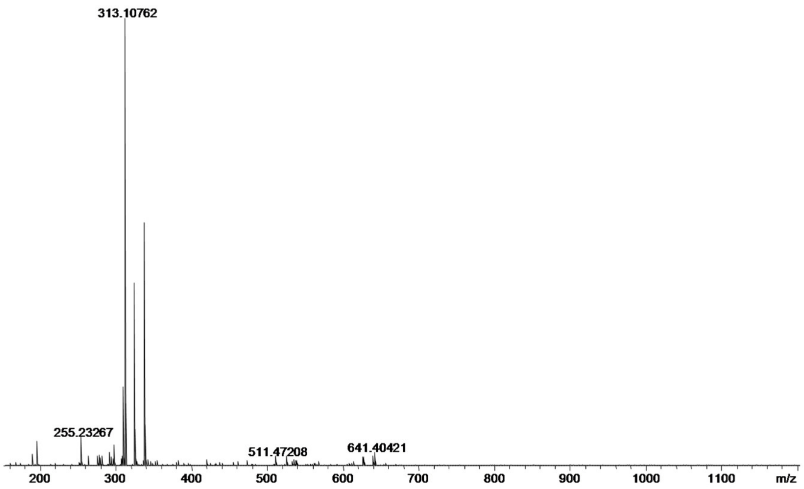
313.10762
255.23267
511.47208
641.40421
200
300
400
500
600
700
800
900
1000
1100
m/z

C)

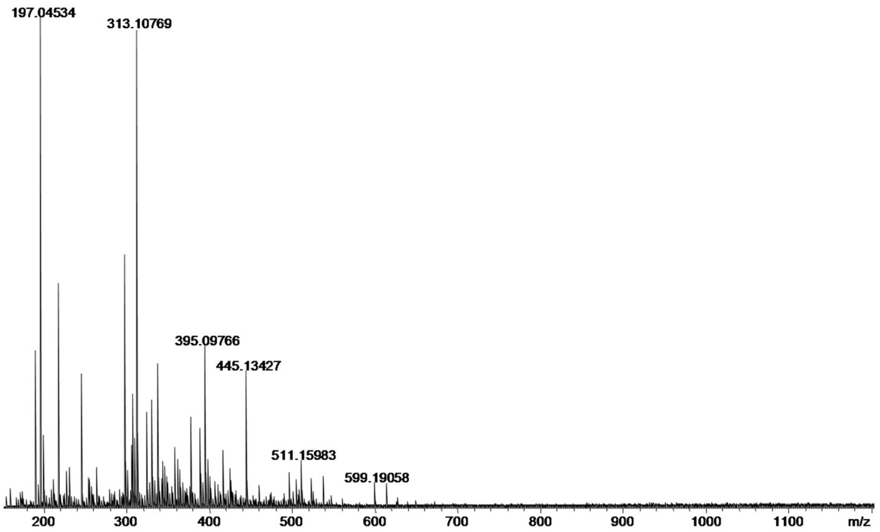

D)

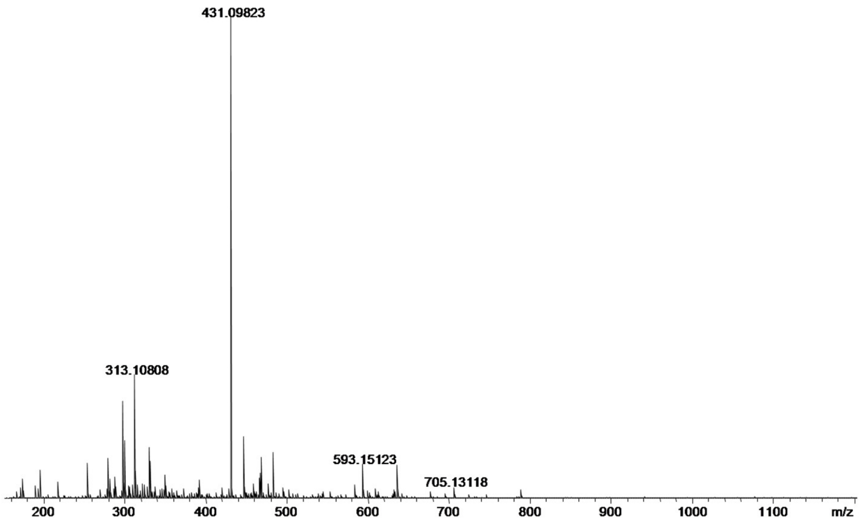

Figure S1: NMR spectrum of crude extract and partitions. The 19 crude extract (A) and partitions , HP (B), CP (C) and AEP (D) were analyzed using the negative ion-mode Electrospray Ionization Fourier Transform Ion Cyclotron Resonance Mass Spectrometer (ESI (−) FT-ICR MS, model 9.4 T Solarix, Bruker Daltonics Bremen). All mass spectra were externally calibrated using NaTFA (*m*/*z* from 200 to 2000). The degree of unsaturation for each molecule can be deduced directly from its DBE value according to the equation DBE = c - h/2 + n/2 + 1, where c, h, and n are the numbers of carbon atoms, hydrogens, and nitrogen in the molecular formula, respectively. The FT-ICR mass spectrum was acquired and processed using Compass Data Analysis software. The elemental compositions of the present compounds were determined by measuring the *m*/*z* ratio values.
